# KCNJ5 Somatic Mutation Is Associated With Higher Aortic Wall Thickness and Less Calcification in Patients With Aldosterone-Producing Adenoma

**DOI:** 10.3389/fendo.2022.830130

**Published:** 2022-03-02

**Authors:** Bo-Ching Lee, Victor Jing-Wei Kang, Chien-Ting Pan, Jia-Zheng Huang, Yu-Li Lin, Yi-Yao Chang, Cheng-Hsuan Tsai, Chia-Hung Chou, Zheng-Wei Chen, Che-Wei Liao, Yu-Wei Chiu, Vin-Cent Wu, Chi-Sheng Hung, Chin-Chen Chang, Yen-Hung Lin

**Affiliations:** ^1^Department of Medical Imaging, National Taiwan University Hospital, Taipei, Taiwan; ^2^Graduate Institute of Clinical Medicine, National Taiwan University College of Medicine, Taipei, Taiwan; ^3^Departments of Medical Imaging, National Taiwan University Hospital Yun-lin Branch, Douliu, Taiwan; ^4^Departments of Internal Medicine, National Taiwan University Hospital Yun-lin Branch, Douliu, Taiwan; ^5^Department of Business Administration and Graduate School of Service Management, Chihlee University of Technology, New Taipei City, Taiwan; ^6^Department of Internal Medicine, National Taiwan University Hospital, Taipei, Taiwan; ^7^Department of Cardiovascular Medicine, Far Eastern Memorial Hospital, New Taipei City, Taiwan; ^8^Department of Obstetrics and Gynecology, National Taiwan University Hospital, Taipei, Taiwan; ^9^Department of Internal Medicine, National Taiwan University Hospital Hsin-Chu Branch, HsinChu, Taiwan; ^10^Department of Computer Science and Engineering, Yuan Ze University, Taoyuan City, Taiwan; ^11^Cardiovascular Center, National Taiwan University Hospital, Taipei, Taiwan

**Keywords:** hypertension, humans, KCNJ5 somatic mutation, primary aldosteronism, aortic calcification

## Abstract

**Objective:**

Primary aldosteronism (PA) is the most common type of secondary hypertension, and it is associated with a higher rate of cardiovascular complications. KCNJ5 somatic mutations have recently been identified in aldosterone-producing adenoma (APA), however their influence on vascular remodeling and injury is still unclear. The aim of this study was to investigate the association between KCNJ5 somatic mutation status and vascular status.

**Methods:**

We enrolled 179 APA patients who had undergone adrenalectomy from a prospectively maintained database, of whom 99 had KCNJ5 somatic mutations. Preoperative clinical, biochemical and imaging data of abdominal CT, including abdominal aortic calcification (AAC) score, aortic diameter and wall thickness at levels of superior (SMA) and inferior (IMA) mesenteric arteries were analyzed.

**Results:**

After propensity score matching for age, sex, body mass index, triglycerides and low-density lipoprotein, there were 48 patients in each KCNJ5 (+) and KCNJ5 (-) group. Mutation carriers had a lower AAC score (217.3 ± 562.2 vs. 605.6 ± 1359.1, P=0.018), higher aortic wall thickness (SMA level: 2.2 ± 0.6 mm vs. 1.8 ± 0.6 mm, P=0.006; IMA level: 2.4 ± 0.6 mm vs. 1.8 ± 0.7 mm, P<0.001) than non-carriers. In multivariate analysis, KCNJ5 mutations were independently associated with AAC score (P=0.014) and aortic wall thickness (SMA level: P<0.001; IMA level: P=0.004). After adrenalectomy, mutation carriers had less aortic wall thickness progression than non-carriers (Δthickness SMA: -0.1 ± 0.8 mm vs. 0.9 ± 0.6 mm, P=0.024; IMA: -0.1 ± 0.6 mm vs. 0.8 ± 0.7 mm, P=0.04).

**Conclusion:**

KCNJ5 mutation carriers had less calcification burden of the aorta, thickened aortic wall, and less wall thickness progression than non-carriers.

## Introduction

Primary aldosteronism (PA) is characterized by abnormal aldosterone hypersecretion, and it is the most common cause of secondary hypertension. Approximately 5-10% of general hypertensive patients may have PA (1), and this rate can be as high as 20% in patients with resistant hypertension (2). Clinically, PA patients are associated with higher cardiovascular events than those with essential hypertension (3, 4).

Excessive aldosterone is related to various cardiovascular injuries. Animal studies have shown that the infusion of aldosterone can cause increased arterial stiffness and vascular fibronectin accumulation, and that the damage can be reversed by an aldosterone antagonist (5). In human studies, higher pulse wave velocity has been reported in PA patients compared to those with essential hypertension, indicating increased arterial stiffness in PA patients (6, 7). These aldosterone-induced cardiovascular injuries may be reversible, as studies have shown that adrenalectomy can ameliorate increased carotid intima-media thickness and arterial stiffness in patients with aldosterone-producing adenoma (APA) (7, 8).

APA is one of the most common subtypes of PA and is surgically correctable (9). KCNJ5 (coding for potassium channel GIRK4) mutations are the most frequently identified somatic mutations in both Western and Asian countries, with a prevalence rate of 34-45% (10–12) and 55-75%, respectively (13–16). APA patients with KCNJ5 mutations tend to be younger, have a higher aldosterone level, lower potassium level, and higher cure rate after adrenalectomy (17). Regarding the effect of KCNJ5 mutations on the cardiovascular system, mutation carriers have been shown to exhibit greater post-operative regression of observed left ventricular remodeling and improvement in arterial stiffness compared to non-carriers (18–20).

Aortic calcification is considered to be an irreversible endpoint of vascular atherosclerosis and an ideal indicator of vascular injury (21), and it can be quantitatively evaluated on CT. Aortic wall thickness of the common carotid artery, thoracic aorta and abdominal aorta is also regarded to be an important marker of vascular atherosclerosis with the ability to predict cardiovascular events (22, 23), and it has been associated with cardiovascular risk factors such as old age, male sex, smoking, elevated systolic blood pressure and low-density lipoprotein.

The aim of this study was to assess the influence of KCNJ5 mutations on atherosclerotic parameters on CT and use propensity score matching (PSM) analysis to balance possible confounding factors.

## Materials and Methods

### Patient Enrollment

In this study, we retrospectively analyzed 179 APA patients who underwent adrenalectomy between September 2006 and March 2019 at National Taiwan University Hospital (NTUH) from a prospectively maintained database. This study was approved by the Institutional Review Board of NTUH, and the need for written informed consent was waived. The clinical information of the patients, including demographic data, atherosclerotic parameters of abdominal CT, and histopathological results of APAs were recorded.

### Laboratory Measurements

Plasma aldosterone concentration (PAC) and plasma renin activity (PRA) were measured using specific radioimmunoassay kits (Aldosterone Maia Kit; Adaltis Italia, Bologna, Italy and DiaSorin, Stillwater, Minnesota, USA, respectively). The aldosterone-to-renin ratio (ARR) was calculated as PAC/PRA.

### Diagnostic Criteria for APA

The diagnosis of APA was established based on the modified four corner criteria as reported previously (19, 24, 25), including: (1) excess aldosterone production confirmed according to an ARR > 35, TAIPAI score > 60%; and seated post-saline loading PAC > 16 ng/dl or urine aldosterone > 12 μg/24 h; (2) identification of adrenal nodules on CT; (3) lateralization of aldosterone hypersecretion by adrenal venous sampling (AVS) or dexamethasone suppression NP-59 single-photon emission CT; (4) pathological evidence of adenoma after adrenalectomy, and subsequent clinical improvement defined by either complete resolution of hypertension or partial resolution of hypertension, potassium, PAC, and PRA.

In this study, successful AVS was defined as a sampled adrenal plasma cortisol concentration (PCC) similar to or two-fold greater than sampled peripheral PCC. The lateralization of PA was determined on the basis of a lateralization index of ≥2.0, which was estimated as the ratio of the sampled adrenal PAC/PCC on the dominant side to the PAC/PCC on the contralateral side.

### Imaging Analysis

Abdominal CT data were available for all APA patients and were obtained using routine techniques (helical CT, 5-mm slices). One radiologist (V.J.K) with 3 years of experience independently evaluated the images on a standard imaging workstation and was unaware of the KCNJ5 mutation status. Aortic wall thickness and diameter were measured at superior mesenteric artery (SMA) and inferior mesenteric artery (IMA) levels, respectively. The thickest portion of the aortic wall and maximum aortic transverse diameter were estimated using reconstructed contrast-enhanced CT images perpendicular to the vascular centerline, to avoid overestimation due to tortuous segments of the aorta. Abdominal aortic calcification (AAC) score was calculated using the CT-based Agatston method from unenhanced abdominal CT axial view images using commercial software (Philips IntelliSpace Portal, Best, Netherlands). Vascular calcifications with attenuation greater than the predefined 130-HU threshold were estimated. A region of interest was manually selected so that only calcifications in the abdominal aorta were included. In this study, we used the Agatston score as described by Agatston for abdominal aorta (26).

### Histopathologic Study and Sequencing of the *KCNJ5* Gene

Laparoscopic adrenalectomy was used for all APA patients in this study. The resected adrenal specimens were blindly inspected by a pathologist. Nodules consisting of adrenal cells and a clearly demarcated pseudo-capsule were defined as adenomas. Before DNA extraction, fresh APA specimens were frozen at −80°C until use. Genomic DNA was prepared using a QIAamp DNA mini kit (Qiagen, Hilden, Germany) for each tissue sample. Exome sequencing was used to assess the coding region of the genomic DNA. Four sets of gene-specific primers were used to amplify and sequence the whole coding sequence (exons 2–3) and the flanking regions of *KCNJ5* (27). The PCR reactions was set at 58°C for primer annealing using GoTaq^®^ Master Mix (Promega Corporation, Madison, USA), and DNA fragments were extracted using a GenepHlow™ Gel/PCR Kit (Geneaid, Taipei, ROC). The PCR products were sent for Sanger sequencing using a 3730 DNA Analyzer (Applied Biosystems, Foster City, USA).

### Statistical Analysis

All statistical analyses were performed using MedCalc statistical software (MedCalc version 15.4.0.0, Frank Schoonjans, Mariakerke, Belgium). Differences between categorical variables were compared using Fisher’s exact test. For independent continuous variables, the differences were compared using an independent two-sample t-test. Continuous variables with skewed distribution such as PAC, ARR, and calcium score were compared using the Mann-Whitney U test. We used propensity score analysis to eliminate possible confounders between the KCNJ5 mutation carrier and non-carrier groups. Clinical variables including age, sex, body mass index (BMI) and low-density lipoprotein (LDL) were included to generate propensity scores. The maximum allowable difference was 8 for age, 6 for BMI, 14 for LDL, and an exact match for sex, with 1:1 matching to select patients from both groups for subsequent analysis. Independent associations between KCNJ5 mutation status and AAC score, aortic wall thickness, aortic diameter progression, aortic wall thickness progression and AAC score progression were investigated using multivariable regression analyses. For all statistical analyses, the significance was 2-tailed with a threshold for significance of P<0.05.

## Results

### Clinical Data in All APA Patients Before and After Matching

Of the 179 APA patients, 99 had KCNJ5 mutations and the other 80 did not. A comparison of the pre-operative clinical variables of the two groups are summarized in **Table 1**. The mutation carriers were younger (P<0.001) and had lower serum levels of potassium (P<0.001) and triglycerides (P<0.001) than the non-carriers. The other clinical variables including sex, BMI, blood pressure, duration of hypertension, and number of hypertensive medications were similar between both groups. After 1:1 PSM for age, sex, BMI and serum triglycerides, each group had 48 patients remained in each group. The matched mutation carriers had a lower serum potassium level (P<0.001) than the non-carriers, while other clinical variables were balanced without significant difference (**Table 1**).

**Table 1:** Demographic characteristics in patient with APA before and after propensity score matching.

	Before propensity score matching			After propensity score matching		
	*KCNJ5(-)* (n=80)	*KCNJ5(+)* (n=99)	*P* value	*KCNJ5(-)* (n=48)	*KCNJ5(+)* (n=48)	*P* value
**Age, year**	54.4 ± 11.4	48.6 ± 9.7	<0.001***	53.6 ± 8.8	51.8 ± 8.3	0.306
**Sex, male**	40 (50.0%)	45 (45.4%)	0.548	23 (47.9%)	22 (45.8%)	0.838
**Height, cm**	164.0 ± 9.3	163.4 ± 8.7	0.671	164.6 ± 8.8	163.3 ± 8.4	0.475
**Weight, kg**	67.8 ± 16.2	66.1 ± 14.8	0.474	68.2 ± 15.2	65.9 ± 13.5	0.436
**BMI, kg/m^2^**	25.0 ± 4.3	24.6 ± 4.0	0.517	25.0 ± 4.1	24.5 ± 3.3	0.515
**SBP, mmHg**	152.9 ± 19.3	154.0 ± 20.9	0.720	150.7 ± 18.3	154.8 ± 20.5	0.311
**DBP, mmHg**	91.0 ± 13.2	94.5 ± 14.8	0.096	91.5 ± 13.1	95.4 ± 13.2	0.149
**HTN years, n**	7.3 ± 6.7	5.8 ± 5.7	0.124	6.8 ± 5.5	6.4 ± 5.4	0.690
**HTN drugs, n**	2.2 ± 1.4	2.2 ± 1.1	0.930	1.9 ± 1.3	1.8 ± 1.1	0.604
**PAC, ng/dL**	53.2 ± 35.7	62.0 ± 41.2	0.133	54.7 ± 36.8	61.7 ± 38.4	0.363
**PRA, ng/mL/h**	0.9 ± 3.2	0.4 ± 0.6	0.093	1.0 ± 3.6	0.5 ± 0.8	0.325
**ARR**	1295 ± 2598	1170 ± 2545	0.746	1283 ± 2705	974 ± 2255	0.544
**Log PAC**	1.63 ± 0.30	1.69 ± 0.29	0.158	1.65 ± 0.29	1.72 ± 0.26	0.240
**Log PRA**	-0.72 ± 0.79	-0.77 ± 0.62	0.631	-0.70 ± 0.79	-0.70 ± 0.63	0.974
**Log ARR**	2.36 ± 0.86	2.47 ± 0.69	0.322	2.35 ± 0.86	2.42 ± 0.66	0.646
**K, mmol/L**	3.8 ± 0.5	3.2 ± 0.6	<0.001***	3.8 ± 0.5	3.2 ± 0.6	<0.001***
**Cr, mg/dL**	1.0 ± 0.4	0.9 ± 0.4	0.258	0.9 ± 0.3	1.0 ± 0.5	0.326
**LDL, mg/dL**	109.8 ± 33.1	101.6 ± 25.7	0.090	105.7 ± 27.5	105.2 ± 24.8	0.926
**TG, mg/dL**	144.4 ± 88.6	104.0 ± 51.5	<0.001***	139.2 ± 77.5	114.8 ± 54.1	0.076
**TC, mg/dL**	189.3 ± 42.9	174.9 ± 31.7	0.018*	184.2 ± 39.0	177.5 ± 23.8	0.319

ARR, aldosterone-to-renin ratio; BMI, body mass index; Cr, creatinine; DBP, diastolic blood pressure; EH, essential hypertension; HTN, hypertension; LDL, low-density lipoprotein cholesterol; PAC, plasma aldosterone concentration; PRA, plasma renin activity; SBP, systolic blood pressure; TG, triglyceride; TC, total cholesterol.

Age, sex, BMI and LDL were matched in propensity score analysis.

*P < 0.05, ***P < 0.001.

### Atherosclerotic Parameters Before and After Matching

The atherosclerotic parameters on abdominal CT, including aortic diameter, aortic wall thickness and AAC score, were evaluated for both groups in this study. Before PSM, the mutation carriers had an increased aortic wall thickness at the SMA (P=0.001) and IMA levels (P=0.001), but a lower rate of mitral valve calcification (P=0.026) and lower AAC score (P<0.001) compared to the non-carriers (**Table 2**). After PSM, the mutation carriers still had a higher aortic wall thickness at the SMA (P=0.006) and IMA levels (P<0.001), but a lower AAC score (P=0.018) than the non-carriers (**Table 2**).

**Table 2:** Abdominal aortic calcification, diameter and thickness in patient with APA before and after propensity score matching.

	Before propensity score matching			After propensity score matching		
	*KCNJ5(-)* (n=80)	*KCNJ5(+)* (n=99)	*P* value	*KCNJ5(-)*(n=48)	*KCNJ5(+)*(n=48)	*P* value
SMA level						
**Diameter, mm**	20.8 ± 2.6	20.4 ± 2.3	0.245	21.1 ± 2.4	20.6 ± 2.3	0.318
**Thickness, mm**	1.8 ± 0.6	2.1 ± 0.6	0.001**	1.8 ± 0.6	2.2 ± 0.6	0.006**
IMA level						
**Diameter, mm**	16.7 ± 2.2	16.6 ± 2.1	0.768	16.8 ± 2.0	16.8 ± 1.9	1.000
**Thickness, mm**	2.0 ± 0.8	2.3 ± 0.6	0.001**	1.8 ± 0.7	2.4 ± 0.6	<0.001***
**MV calcification, n**	9/80	2/98	0.026*	3/48	1/48	0.307
**AAC**	796.7 ± 1607.3	160.4 ± 432.5	<0.001***	605.6 ± 1359.1	217.3 ± 562.2	0.018*

AAC, abdominal aortic calcification; IMA, inferior mesenteric artery; MV, mitral valve; SMA, superior mesenteric artery.

*P < 0.05, **P < 0.01, ***P < 0.001.

### Factors Associated With Baseline Aortic Wall Thickness

In univariate analysis (**Table 3**), aortic wall thickness at the SMA level was associated with KCNJ5 mutations (P=0.001) and the duration of hypertension (P=0.044), and aortic wall thickness at the IMA level was associated with KCNJ5 mutations (P=0.001), potassium (P=0.034) and creatinine (P=0.002) levels. In multivariable analysis, KCNJ5 mutations were independently associated with aortic wall thickness at the SMA (β=0.279, P<0.001) and IMA (β=0.251, P=0.004) levels.

**Table 3:** Multivariable regression analyses for factors associated with the aortic wall thickness.

	Thickness (SMA level)				Thickness (IMA level)			
	Univariate		Multivariate		Univariate		Multivariate	
	β coefficient	*P* value	β coefficient	*P* value	β coefficient	*P* value	β coefficient	*P* value
**KCNJ5 mutations**	0.254	0.001**	0.279	<0.001***	0.248	0.001**	0.251	0.004**
**Age, year**	0.108	0.170			0.042	0.590		
**Sex, male**	0.084	0.289			-0.088	0.263		
**Height, cm**	-0.075	0.340			0.105	0.181		
**Weight, kg**	-0.038	0.628			0.095	0.228		
**BMI, kg/m^2^**	-0.002	0.977			0.061	0.442		
**SBP, mmHg**	0.013	0.871			0.075	0.338		
**DBP, mmHg**	-0.067	0.398			0.015	0.849		
**HTN years, n**	0.158	0.044*	0.193	0.012*	0.029	0.717		
**HTN drugs, n**	0.083	0.296			0.125	0.113		
**Log PAC**	0.049	0.537			0.138	0.080		
**Log PRA**	-0.022	0.778			-0.021	0.793		
**Log ARR**	0.039	0.621			0.071	0.366		
**K, mmol/L**	-0.148	0.060			-0.167	0.034*	-0.043	0.620
**Cr, mg/dL**	0.011	0.891			0.244	0.002**	0.268	<0.001***
**LDL, mg/dL**	-0.071	0.413			-0.129	0.136		
**TG, mg/dL**	0.007	0.934			-0.003	0.967		
**TC, mg/dL**	-0.076	0.364			-0.089	0.284		

Factors with P value less than 0.05 were selected into multivariable regression analyses. ARR, aldosterone-to-renin ratio; BMI, body mass index; Cr, creatinine; DBP, diastolic blood pressure; EH, essential hypertension; HTN, hypertension; LDL, low-density lipoprotein cholesterol; PAC, plasma aldosterone concentration; PRA, plasma renin activity; SBP, systolic blood pressure; TG, triglyceride; TC, total cholesterol.

*P < 0.05, **P < 0.01, ***P < 0.001.

### Factors Associated With Baseline Aortic Calcification

As shown in **Table 4**, AAC score was related to several factors including KCNJ5 mutations (P<0.001), age (P<0.001), systolic blood pressure (P=0.020), duration of hypertension (P=0.001) and number of hypertension drugs (P=0.003) in univariate analysis. In multivariable analysis, AAC score was independently associated with KCNJ5 mutations (β=-0.168, P=0.014), age (β=0.387, P<0.001), systolic blood pressure (β=0.171, P=0.010), and number of hypertension drugs (β=0.153, P=0.024).

**Table 4:** Multivariable regression analyses for factors associated with AAC.

	Univariate		Multivariate	
	β coefficient	*P* value	β coefficient	*P* value
**KCNJ5 mutations**	-0.273	<0.001***	-0.168	0.014*
**Age, year**	0.433	<0.001***	0.387	<0.001***
**Sex, male**	-0.09	0.232		
**Height, cm**	0.024	0.750		
**Weight, kg**	0.037	0.629		
**BMI, kg/m^2^**	0.041	0.588		
**SBP, mmHg**	0.174	0.020*	0.171	0.010*
**DBP, mmHg**	-0.068	0.369		
**HTN years, n**	0.243	0.001**	-0.026	0.741
**HTN drugs, n**	0.223	0.003**	0.153	0.024*
**Log PAC**	0.057	0.451		
**Log PRA**	-0.003	0.966		
**Log ARR**	0.025	0.740		
**K, mmol/L**	0.082	0.274		
**Cr, mg/dL**	0.094	0.210		
**LDL, mg/dL**	-0.018	0.824		
**TG, mg/dL**	0.063	0.424		
**TC, mg/dL**	0.012	0.879		

Factors with P value less than 0.05 were selected into multivariable regression analyses. AAC, abdominal aortic calcification; ARR, aldosterone-to-renin ratio; BMI, body mass index; Cr, creatinine; DBP, diastolic blood pressure; EH, essential hypertension; HTN, hypertension; LDL, low-density lipoprotein cholesterol; PAC, plasma aldosterone concentration; PRA, plasma renin activity; SBP, systolic blood pressure; TG, triglyceride; TC, total cholesterol.

*P < 0.05, **P < 0.01, ***P < 0.001.

### Changes in Atherosclerotic Parameters on Abdominal CT After Adrenalectomy

Twenty-six patients had post-operative CT images. After adjusting for age, sex, and follow-up examination interval in multivariable analysis, the non-carriers had higher aortic wall thickness progression after adrenalectomy at the SMA (Δthickness: -0.1 ± 0.8 mm vs. 0.9 ± 0.6 mm, P=0.024) and IMA (Δthickness: -0.1 ± 0.6 mm vs. 0.8 ± 0.7 mm, P=0.040) levels than the carriers (**Table 5**). The progression of AAC score was similar between the two groups (P=0.732).

**Table 5:** Change of aortic calcification, diameter and thickness in patient with APA before and after adrenalectomy.

	*KCNJ5(-)* (n=15)	*KCNJ5(+)* (n=11)	Adjusted *P* value
SMA level			
**ΔDiameter, mm**	0.1 ± 0.8	0.6 ± 0.5	0.247
**ΔThickness, mm**	0.9 ± 0.6	-0.1 ± 0.8	0.024*
IMA level			
**ΔDiameter, mm**	0.1 ± 1.0	-0.2 ± 1.0	0.195
**ΔThickness, mm**	0.8 ± 0.7	-0.1 ± 0.6	0.040*
**ΔAAC**	359.0 ± 662.8	195.6 ± 384.8	0.732

P value was adjusted for age, sex, and exam interval.

AAC, abdominal aortic calcification; IMA, inferior mesenteric artery; SMA, superior mesenteric artery.

*P < 0.05.

## Discussion

There are several major findings in this study. First, APA patients with KCNJ5 somatic mutations had a thicker aortic wall and less aortic calcification compared to those without KCNJ5 mutations, even after matching for age, sex and blood pressure by PSM. Second, in multivariate analysis, the presence of KCNJ5 mutations was an independent factor associated with aortic wall thickness and aortic calcification. Third, after adrenalectomy, the patients with KCNJ5 mutations had less progression of aortic wall thickness compared to those without KCNJ5 somatic mutations.

The wall of the aorta is composed of tunica intima, tunica media, and tunica adventitia (28). The intima and media of the aortic wall can thicken due to adaptive collagen redistribution from aging, hypertension and pathological atherosclerosis with the formation of fat streaks and plaques (29, 30), and this has been shown to be a good marker of atherosclerosis and coronary artery disease (31, 32). Excessive aldosterone induces chronic inflammation of the vessel walls by increasing reactive oxygen species and proinflammatory transcription factors production (33). Enhanced monocytes and macrophages infiltration and adhesion on the endothelium are noted under high concentration of aldosterone (34), and the infiltrated inflammatory cells again worsen vascular inflammation (35). Aldosterone also promotes vascular remodeling of small arteries by increased collagen, fibronectin and ICAM-1 deposition in the vessel wall (36). PA is clinically associated with increased intima–media thickness of the carotid artery (37, 38), which may regress after adrenalectomy or spironolactone treatment. In this study, the APA patients with KCNJ5 somatic mutations had a thicker abdominal aorta compared to those without KCNJ5 mutations, which is probably due to higher blood pressure and more severe aldosteronism in the mutation carriers (19). In our previous study, APA patients with KCNJ5 somatic mutations had a higher degree of left ventricular hypertrophy and worse diastolic function (19). These findings may also have been due to similar reasons.

During the atherosclerotic process, atheroma or fibrous fatty plaques are formed, followed by calcium deposition in the latter stages (39). In the present study, we found more calcified plaques in those without KCNJ5 somatic mutations. The mechanism of aortic calcification formation is complex and involves multifactorial vascular inflammation (40). In our prior study we investigated serum CRP levels in PA patients, and showed that KCNJ5 mutations were associated with lower levels of pro-inflammation factors (41), metabolic syndrome and abdominal obesity (42). This may explain why the patients without KCNJ5 somatic mutations had a higher burden of aortic calcified plaques despite lower blood pressure and less severe aldosteronism compared to those with KCNJ5 somatic mutations.

Several somatic mutations including *ATP1A1*, *ATP2B3*, *CACNA1D* and *KCNJ5* may contribute to the pathogenesis of APA (43–47). These mutations activate calcium signaling and lead to aldosterone production by increasing the expression of aldosterone synthase. In APA patient with KCNJ5 mutations, opened calcium channels and the overexpression of CYP11B2 lead to excessive aldosterone secretion from the adrenal tumor (47, 48). These mutation carriers have been found to have more severe aldosteronism compared to non-carriers (11, 12). Recently, CYP11B2 immunohistochemistry (IHC)-guided biopsy has been shown to increase the diagnostic performance of somatic mutations to around 87-94% in APA patients (49–51). One study demonstrated that an IHC-guided biopsy could increase the detection of somatic mutations in frozen adenoma tissue from 71% to 94% compared to a random biopsy by Sanger sequencing (51). In the present study, a random biopsy was used to sequence KCNJ5 mutations without the aid of CYP11B2 IHC guidance. However, an IHC-guided biopsy may have limited value in detecting KCNJ5 mutations, as they could be detected regardless of CYP11B2 expression in a random biopsy. Thus, we believe that the rate of KCNJ5 mutations identified by random biopsy is reliable in our study. Notably, the prevalence of other somatic mutations besides KCNJ5 were low (<6%) in our prior studies (13), indicating that the detection performance for other somatic mutations may be inferior by random biopsy compared to IHC-guided biopsy. As a result, the non-carriers of KCNJ5 mutations in the present study may have had various types of somatic mutations besides KCNJ5. However, we focused on the influence of KCNJ5 mutations with regards to aortic atherosclerosis in this study; therefore, our conclusions may not be affected by the lack of IHC-guided biopsy.

There are several limitations to this study. First, somatic mutations besides KCNJ5 such as ATP1A1, ATP2B3, CACNA1D and other new genes were not evaluated in this study, and the patients were grouped as being non-carriers of KCNJ5 mutations (44, 46, 52, 53). In addition, we did not use CYP11B2 IHC-guided biopsy to detect somatic mutations, which may have performed better than random biopsy. Second, despite the use of PSM to ameliorate imbalances in age, sex, BMI and LDL between the two groups of APA patients, other unknown discrepancies may have contributed to the increased aortic calcification in the non-carriers, and we do not have pathological data regarding the atherosclerotic burden of resected adrenal samples. Third, the number of patients with available follow-up abdominal CT was relatively small (n=26). However, the progression of aortic wall thickness remained significant after adjusting for age, sex and examination interval, indicating that small number of patients only had a limited impact. Finally, the CT images used to generate AAC and aortic wall thickness used 5.0 mm slice reconstruction. Although the correlation between the measured AAC from 2.5mm and 5.0mm slice thickness was almost perfect (r = 0.999, P < 0.0001), a 10% underestimation of AAC maybe expected due to partial volume effect (54, 55).

In conclusion, APA patients with KCNJ5 somatic mutations had a thicker abdominal aortic wall but less atherosclerotic calcification compared to those without KCNJ5 mutations. After surgery, the APA patients with KCNJ5 somatic mutations had less wall thickness progression than the non-carriers.

## Data Availability Statement

The raw data supporting the conclusions of this article will be made available by the authors, without undue reservation.

## Ethics Statement

The studies involving human participants were reviewed and approved by Institutional Review Board of NTUH. Written informed consent for participation was not required for this study in accordance with the national legislation and the institutional requirements.

## Author Contributions

B-CL: project concept and design, data collection, imaging analysis, data analysis, and write up. VK: data collection, imaging analysis, and write up. C-CC project concept and design. J-ZH: data collection, imaging analysis. Y-YC: project concept and design. C-HT: project concept and design. Z-WC: project concept and design. Y-LL: project concept and design. C-HC: critical revisions. C-WL: critical revisions. C-TP: critical revisions. C-SH: critical revisions. V-CW: project concept and design, data collection, and critical revisions. Y-HL: project concept and design, data collection, imaging analysis, and critical revisions. All authors contributed to the article and approved the submitted version.

## Appendix

Membership of the Taiwan Primary Aldosteronism Investigation (TAIPAI) Study Group: Che-Hsiung Wu, MD (Chi-Taz Hospital, PI of Committee); V-CW, MD (NTUH, PI of Committee); Y-HL, MD (NTUH, PI of Committee); Hung-Wei Chang, MD, PhD (Far Eastern Clinics, PI of Committee); Lian-Yu Lin MD, PhD (NTUH, PI of Committee); Fu-Chang Hu, MS, ScD, (Harvard Statistics, Site Investigator); Kao-Lang Liu, MD (NTUH, PI of Committee); Shuo-Meng Wang, MD (NTUH, PI of Committee); Kuo-How Huang, MD (NTUH, PI of Committee); Yung-Ming Chen, MD (NTUH, PI of Committee); C-CC, MD (NTUH, PI of Committee); Shih-Cheng Liao, MD (NTUH, PI of Committee); Ruoh-Fang Yen, MD, PhD (NTUH, PI of Committee); and Kwan-Dun Wu, MD, PhD (NTUH, Director of Coordinating Center).

## Funding

This study was supported by National Taiwan University Hospital (NTUH 108-A141), Ministry of Science and Technology (MOST 106-2314-B-002-169-MY3), and Department of Health, Executive Yuan, R.O.C. (PTH10744). The funders had no role in study design, data collection and analysis, decision to publish, or preparation of the manuscript.

## Conflict of Interest

The authors declare that the research was conducted in the absence of any commercial or financial relationships that could be construed as a potential conflict of interest.

## Publisher’s Note

All claims expressed in this article are solely those of the authors and do not necessarily represent those of their affiliated organizations, or those of the publisher, the editors and the reviewers. Any product that may be evaluated in this article, or claim that may be made by its manufacturer, is not guaranteed or endorsed by the publisher.
